# Oleanolic Acid: A Novel Cardioprotective Agent That Blunts Hyperglycemia-Induced Contractile Dysfunction

**DOI:** 10.1371/journal.pone.0047322

**Published:** 2012-10-16

**Authors:** Rudo F. Mapanga, Uthra Rajamani, Nonkululeko Dlamini, Makhosazane Zungu-Edmondson, Roisin Kelly-Laubscher, Mohammed Shafiullah, Athiq Wahab, Mohamed Y. Hasan, Mohamed A. Fahim, Philippe Rondeau, Emmanuel Bourdon, M. Faadiel Essop

**Affiliations:** 1 Cardio-Metabolic Research Group (CMRG), Department of Physiological Sciences, Stellenbosch University, Stellenbosch, South Africa; 2 Discipline of Physiology, School of Medical Sciences, University of KwaZulu-Natal, Durban, South Africa; 3 Department of Human Biology, Faculty of Health Sciences, University of Cape Town, Observatory, South Africa; 4 Faculty of Medicine and Health Sciences, United Arab Emirates University, Al-Ain, United Arab Emirates; 5 Groupe d’Etude sur l’Inflammation Chronique et l’Obésité (GEICO), Université de La Réunion, Saint Denis de La Réunion, France; Virginia Commonwealth University Medical Center, United States of America

## Abstract

Diabetes constitutes a major health challenge. Since cardiovascular complications are common in diabetic patients this will further increase the overall burden of disease. Furthermore, stress-induced hyperglycemia in non-diabetic patients with acute myocardial infarction is associated with higher in-hospital mortality. Previous studies implicate oxidative stress, excessive flux through the hexosamine biosynthetic pathway (HBP) and a dysfunctional ubiquitin-proteasome system (UPS) as potential mediators of this process. Since oleanolic acid (OA; a clove extract) possesses antioxidant properties, we hypothesized that it attenuates acute and chronic hyperglycemia-mediated pathophysiologic molecular events (oxidative stress, apoptosis, HBP, UPS) and thereby improves contractile function in response to ischemia-reperfusion. We employed several experimental systems: 1) H9c2 cardiac myoblasts were exposed to 33 mM glucose for 48 hr vs. controls (5 mM glucose); and subsequently treated with two OA doses (20 and 50 µM) for 6 and 24 hr, respectively; 2) Isolated rat hearts were perfused *ex vivo* with Krebs-Henseleit buffer containing 33 mM glucose vs. controls (11 mM glucose) for 60 min, followed by 20 min global ischemia and 60 min reperfusion ± OA treatment; 3) *In vivo* coronary ligations were performed on streptozotocin treated rats ± OA administration during reperfusion; and 4) Effects of long-term OA treatment (2 weeks) on heart function was assessed in streptozotocin-treated rats. Our data demonstrate that OA treatment blunted high glucose-induced oxidative stress and apoptosis in heart cells. OA therapy also resulted in cardioprotection, i.e. for *ex vivo* and *in vivo* rat hearts exposed to ischemia-reperfusion under hyperglycemic conditions. In parallel, we found decreased oxidative stress, apoptosis, HBP flux and proteasomal activity following ischemia-reperfusion. Long-term OA treatment also improved heart function in streptozotocin-diabetic rats. These findings are promising since it may eventually result in novel therapeutic interventions to treat acute hyperglycemia (in non-diabetic patients) and diabetic patients with associated cardiovascular complications.

## Introduction

The dramatic surge in diabetes during the past few decades constitutes a major threat to human health in developed and developing nations [1], [2]. Since cardiovascular complications and mortalities are common in diabetic patients [3], [4], this will further increase the overall burden of disease. These alarming projections therefore necessitate a comprehensive understanding of the underlying molecular mechanisms orchestrating the onset of cardiovascular diseases (CVD) in diabetic individuals.

Diabetes is characterized by perturbed metabolic pathways usually resulting in hyperlipidemia, hyperinsulinemia and hyperglycemia. Cardiovascular complications frequently present in diabetic patients and chronic hyperglycemia is an important risk factor for myocardial infarction [5], [6]. Moreover, stress-induced, acute hyperglycemia in non-diabetic patients with acute myocardial infarction is associated with increased in-hospital deaths [7], [8]. Acute and chronic hyperglycemia trigger biochemical and electrophysiological changes that may result in impaired cardiac contractile function [9]. Moreover, hyperglycemia also generates reactive oxygen species (ROS) and cell death in the myocardium, thereby contributing to the onset of CVD [10]–[13]. For example, we previously found that hyperglycemia-induced ROS increased flux through the hexosamine biosynthetic pathway (HBP) leading to greater *O*-GlcNAcylation of target proteins and myocardial apoptosis [13], [14]. Hyperglycemia-induced oxidative stress can also result in the formation of misfolded or damaged proteins that may be eliminated by the ubiquitin-proteasome system (UPS). Previous studies revealed dysfunctional UPS with hyperglycemia, linked to greater inflammation and attenuated cardiac function at baseline and in response to ischemia-reperfusion [10], [15]. However, it remains unclear whether increased or decreased UPS is detrimental with hyperglycemia and/or in response to ischemia-reperfusion. For example, Pye et al. [16] found that myocardial reperfusion injury is reduced by proteasomal inhibitors, while others determined that UPS overactivity may enhance the risk of complication during myocardial ischemia in diabetic patients [10]. Conversely, Bulteau et al. [17] established that proteasomal impairment may contribute to the detrimental effects of myocardial ischemia. Additional studies are therefore required to determine the mechanisms underlying dysfunctional UPS in the heart under these conditions.

**Figure 1:**
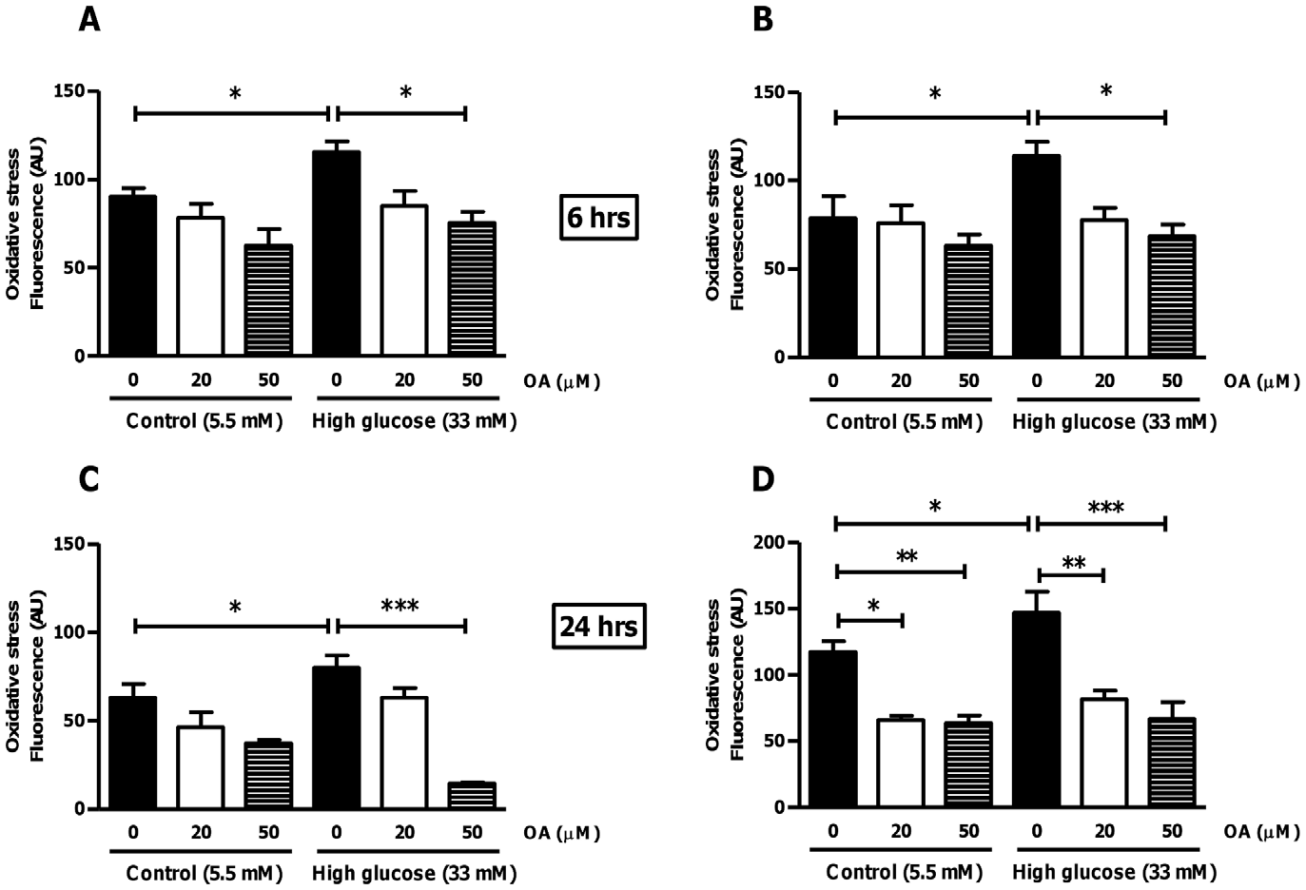
OA treatment attenuates oxidative stress in H9c2 cells. Quantification of oxidative stress (DCFDA staining) in H9c2 cells in response to simulated chronic hyperglycemia (33 mM glucose) vs. control (5.5 mM glucose) ± treatment with 20 µM or 50 µM OA for 6 and 24 hr, respectively. (A) and (C) Fluorescence microscopy; (B) and (D) Flow cytometry. Values are expressed as mean ± SEM (n = 9). *p<0.05, **p<0.01, ***p<0.001 vs. respective controls.

Despite the prevalence of commercially-available drugs used to treat diabetes, the use of alternative, plant-derived medicines is gaining momentum [18]. For example, earlier studies evaluated the anti-diabetic therapeutic potential of *Syzygium aromaticum* [(Linnaeus) Merrill & Perry], belonging to the family Myrtaceae (commonly referred to as cloves). Here research workers established that its active constituent is the triterpenoid, oleanolic acid (OA), displaying anti-hyperglycemic properties [19]–[21]. Furthermore, OA exhibited cardioprotective properties in response to ischemia-reperfusion by upregulation of myocardial anti-oxidant defenses [22], [23]. In light of this, we hypothesized that OA possesses anti-oxidant and anti-apoptotic properties and is thus able to blunt acute and chronic hyperglycemia-mediated pathophysiologic sequelae within the rat heart. Moreover, we proposed that OA attenuates the myocardial UPS and HBP, and thereby improves cardiac contractile function in response to ischemia-reperfusion under hyperglycemic conditions.

**Figure 2:**
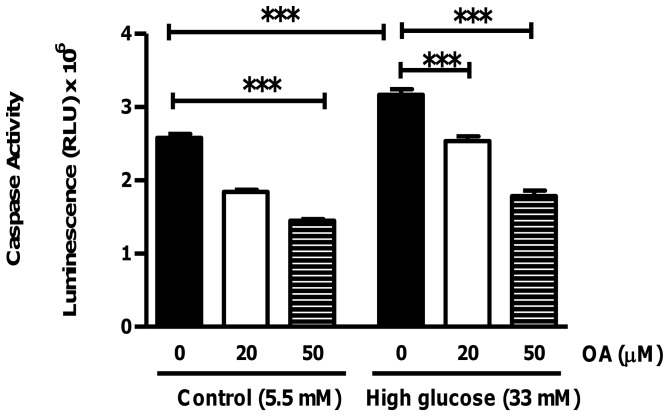
Decreased apoptotic cell death in H9c2 cells treated with OA (caspase activity). Evaluation of caspase activity in H9c2 cells in response to simulated chronic hyperglycemia vs. control ± treatment with 20 µM and 50 µM OA, respectively, for 24 hr. Values are expressed as mean ± SEM (n = 9). ***p<0.001 vs. respective controls.

## Materials and Methods

### Isolation of Oleanolic Acid from Clove Extract

We employed *Syzygium aromaticum* [(Linnaeus) Merrill & Perry] (Myrtaceae) cloves (Africa International Food and Cosmetics Technologies, Durban, South Africa) to isolate and purify OA for this study. This approach was adopted since it generates sufficient amounts of OA in a cost-effective manner compared to purchasing purified OA on a regular basis. Cloves (1 kg) were extracted at room temperature for 24 hr sequentially in 3 liters of each, dichloromethane and ethyl acetate. This step was repeated 3 times to yield residues of dichloromethane-solubles and ethyl acetate-solubles (EAS), respectively. Previous studies demonstrated that OA is mostly concentrated in the latter fraction [21]. Subsequently, filtration was performed with 30 cm filter paper (Whatman International Ltd, Maidstone, England) where after filtrates were concentrated *in vacuo* using a rotary evaporator (Boeco, Hamburg, Germany) at 60°C. This procedure resulted in the isolation of a crude ethyl-acetate extract.

To identify chemical constituents, crude ethyl-acetate extracts were thereafter analyzed by thin layer chromatography (TLC) on pre-coated aluminium plates using Silica Gel 60 F254 (Merck, Darmstadt, Germany). Here we spotted a diluted portion of the isolated, crude extract and compared this with commercially obtained OA (Sigma-Aldrich, St Louis, MO). After developing the TLC plate with ethyl acetate/hexane (7∶3), it was exposed to ultraviolet light (254–366 nm), sprayed with anisaldehyde/sulphuric acid/alcohol solution and the TLC plate subsequently dried with hot air. The appearance of a blue/violet-blue coloration indicated the presence of triterpenoids [23], [24].

**Figure 3:**
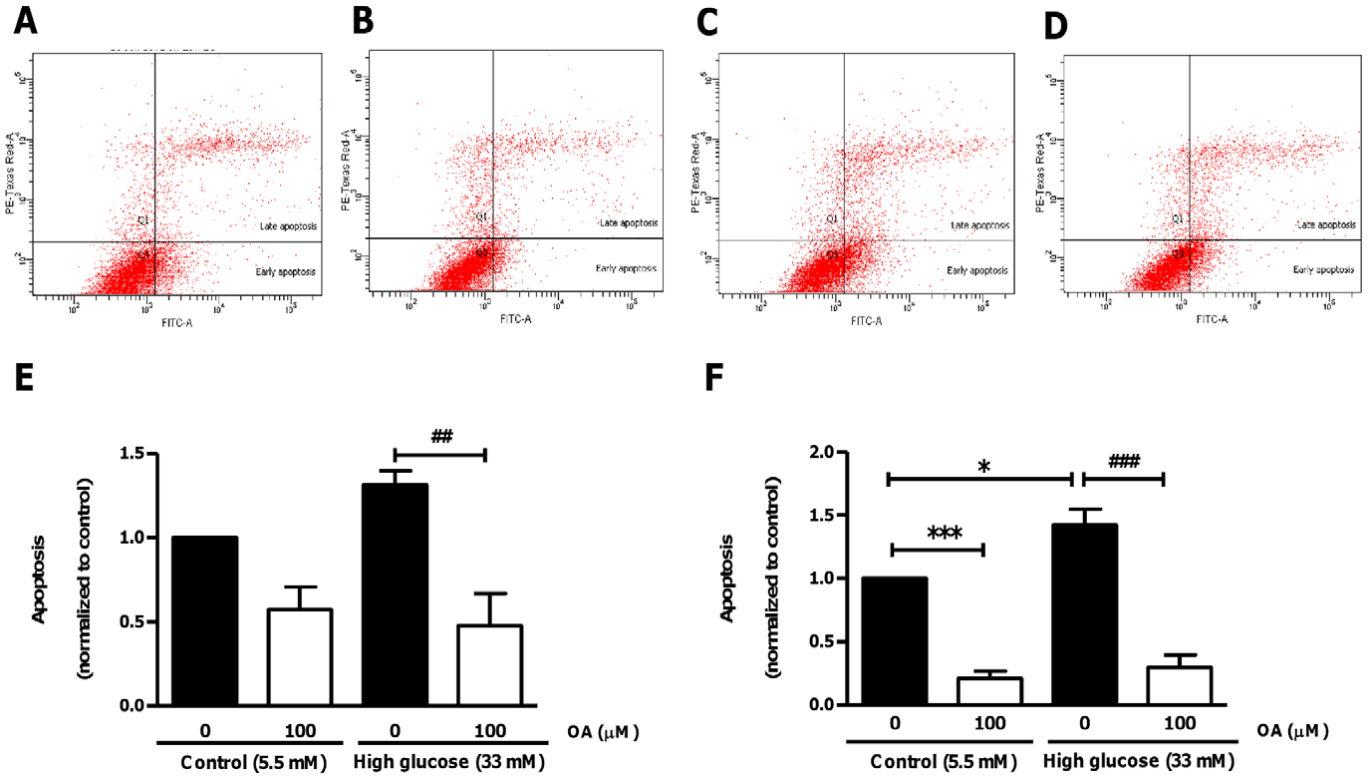
Diminished apoptosis in OA-treated H9c2 cells (flow cytometry). Flow cytometric analysis using the Annexin V/FITC apoptosis assay kit to evaluate the effects of 100 µM OA treatment under control and high glucose culturing conditions (24 hr). (A), (B), (C) and (D) Representative FACS analyses of four individual experiments corresponding to control and high glucose ± OA treatment, respectively. (E) and (F) Quantification of OA treatment after 6 and 24 hr, respectively. Values are normalized to the control and expressed as mean ± SEM (n = 4). ***p<0.001 vs. controls and ##, ### p<0.01, p<0.001 vs. high glucose exposure without OA treatment.

Since the EAS fraction of *S. aromaticum* contained triterpenoids, it was subjected to further purification processes. We fractionated 2 g of EAS on silica gel (70–230 mesh, 3.5×45 cm) by open column chromatography with a ratio of 7∶3 ethyl acetate and hexane, respectively. An aliquot of each collected fraction was then subjected to TLC as before, and compared to commercially obtained OA. This allowed us to pool the remainder of collected fractions according to TLC profiles (i.e. similar to OA), which was thereafter concentrated *in vacuo* using a rotary evaporator (Boeco, Hamburg, Germany) at 55°C. Concentrates were reconstituted using minimal amounts of chloroform and crystallized OA allowed to air dry. We re-crystallized OA with ethanol and its structure was confirmed by spectroscopic analysis using 1D and 2D ^1^H and ^13^C nuclear magnetic resonance techniques to a purity of ∼98%. For a small part of this study we also employed commercially available OA (Sigma-Aldrich, St. Louis, MO) due to logistic reasons.

**Figure 4:**
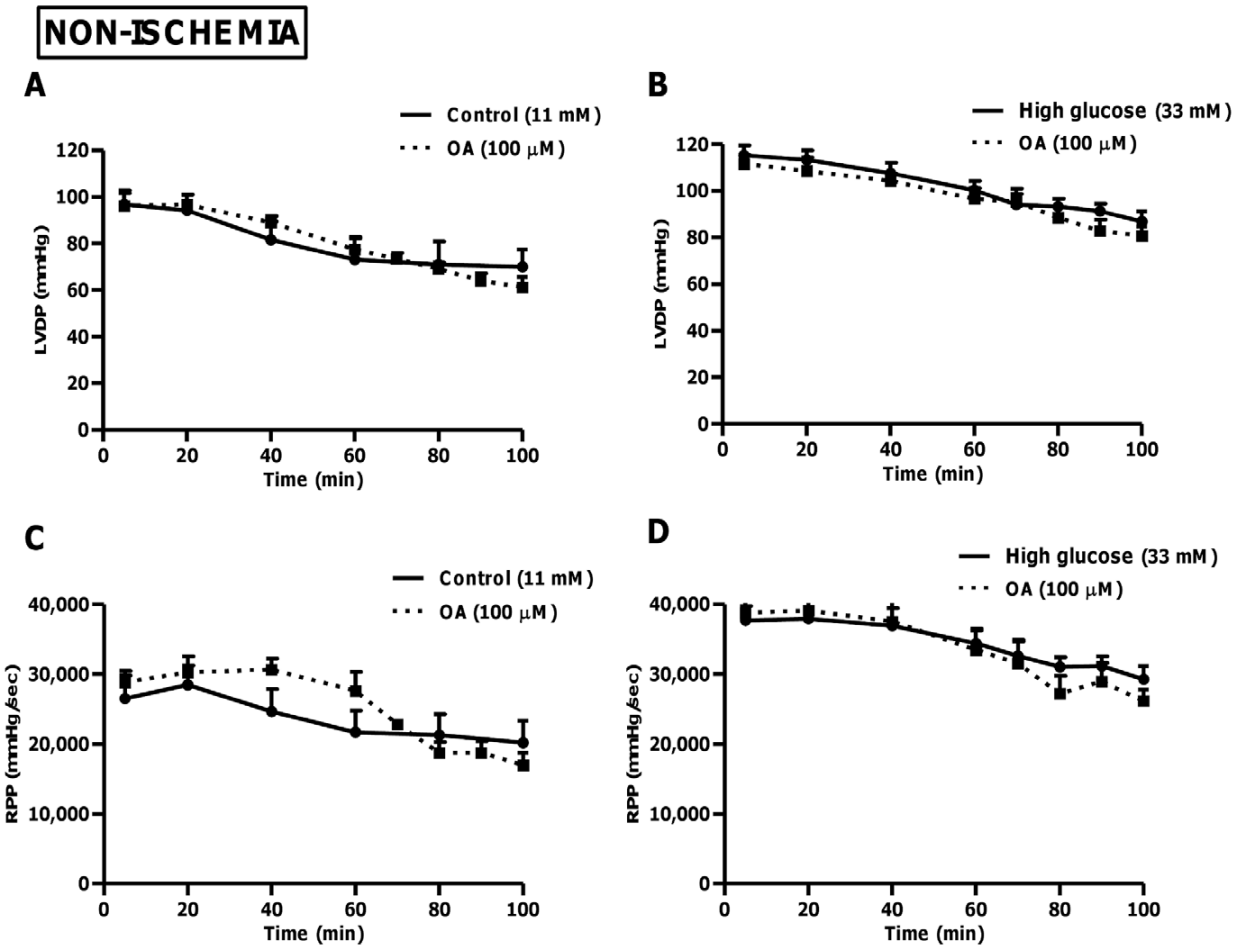
OA treatment does not affect pre-ischemic cardiac function. Isolated rat hearts were perfused under simulated hyperglycemic conditions (33 mM glucose) vs. controls (11 mM glucose) ±100 µM OA treatment. We initially perfused for 60 min, whereafter OA was added for a further 20 min. Subsequently, the buffer initially used was returned and hearts perfused for an additional 20 min. (A) Left ventricular developed pressure at baseline (11 mM) and (B) high glucose conditions (33 mM). Rate pressure product (RPP) at baseline (C) and (D) high glucose levels. Values are expressed as mean ± SEM (n = 9).

### Cell Culture and Oleanolic Acid Treatments

H9c2 rat cardiomyoblasts (ECACC No. 88092904) were maintained at 37°C (5% CO_2_ and 95% humidity) in low glucose (5.5 mM) Dulbecco’s modified Eagle’s medium (DMEM) (Sigma-Aldrich, St. Louis, MO) supplemented with 10% fetal bovine serum (Invitrogen, Carlsbad, CA) as described before by us [13]. On the first day, H9c2 cells were split, sub-cultured and allowed to plate for 24 hr. Cells were thereafter cultured in DMEM containing: 5.5 mM glucose (control group), or 33 mM glucose (high glucose). With the high glucose exposure we attempted to simulate chronic hyperglycemia in our cell-based studies. H9c2 cells were cultured for an additional 48 hr under these conditions followed by dose-dependent treatment with 0, 20, 50 µM OA for 6 and 24 hr, respectively. The doses were selected based on literature [25], [26].

**Figure 5:**
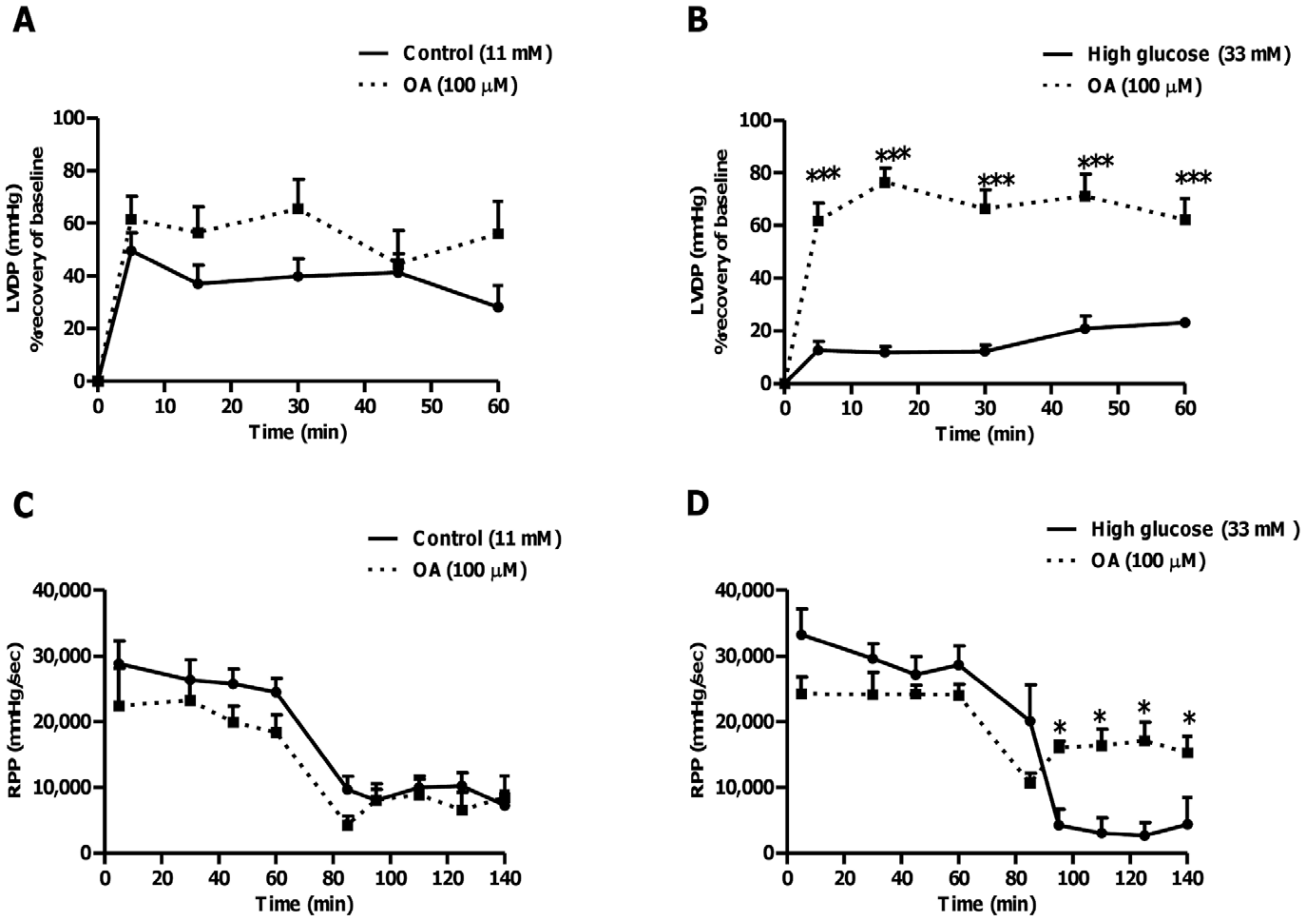
OA treatment blunts high glucose-induced cardiac dysfunction following ischemia and reperfusion. Isolated rat hearts were perfused under simulated hyperglycemic conditions (33 mM glucose) vs. controls (11 mM glucose) and subjected to 20 min of global ischemia, followed by 60 min of reperfusion. For OA treatments groups, 100 µM OA was added during the first 20 min of reperfusion. (A) Left ventricular developed pressure (% recovery) at baseline glucose levels (11 mM), and (B) with high glucose (33 mM). Rate pressure product (RPP) at baseline glucose levels (C), and (D) under high glucose conditions. Values are expressed as mean ± SEM (n = 9).*p<0.05, ***p<0.001 vs. respective controls.

### Measurement of Intracellular ROS Levels and Apoptosis

Intracellular ROS levels were determined by immunofluorescence microscopy as previously described [13]. Briefly, cells were grown in special chamber slides and treated with OA as described above. Subsequently, live cells were incubated with 2′,7′-dichlorodihydrofluorescein diacetate (DCFDA) stain (1∶200; Invitrogen, Carlsbad, CA) for 10 min at 37°C (in the dark). The cells were then further stained with Hoechst dye in PBS at a ratio of 1∶200 for 3–5 min. Stains were then washed off, and cells were visualized using an Olympus CellˆR fluorescence 1×81 inverted microscope (Olympus Biosystems, Planegg, Germany) with an F-view II camera for image acquisition and CellˆR software for processing images. The temperature of the microscope was maintained at 37°C for live cell imaging using a Solent Scientific microscope incubator chamber (Solent Scientific, Segensworth, UK). Three independent experiments were conducted and at least 3 images per experiment analyzed.

**Figure 6:**
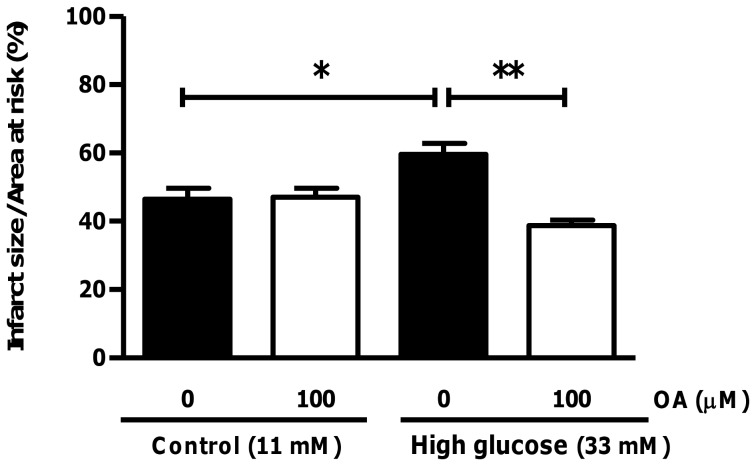
OA administration decreases infarct size under high glucose perfusion conditions. Isolated rat hearts were perfused under high glucose conditions vs. controls and subjected to regional ischemia. For OA treated groups, 100 µM OA was added during the first 20 min of the two hr reperfusion period. Evans blue dye and TTC staining enabled visualization of viable tissue (blue), infarcted area (white) and the area at risk (red). Values are expressed as mean ± SEM (n = 6). *p<0.05, ***p<0.001 vs. respective controls.

We also measured ROS levels by flow cytometry. After treatment, cells were trypsinized and centrifuged (2 min at 20,000 *g*), and the cell pellet treated with 2′,7′-dichlorodihydrofluorescein diacetate stain (1∶200), resuspended, and incubated at 37°C for 20 min in the dark. Cells not treated with 2′,7′-dichlorodihydrofluorescein diacetate acted as negative controls, and stained cells treated with 100 µl hydrogen peroxide (30% w/v hydrogen peroxide) incubated for 10 min served as positive controls. ROS levels were measured using a flow cytometer (Becton-Dickinson, Franklin Lakes, NJ) and quantified by determining the mean of fluorescence for each treatment. Three independent experiments were conducted for each condition investigated, with typically 5,000–10,000 cells analyzed per experiment.

In a separate set of experiments, we evaluated apoptosis by employing a caspase activity assay (Promega, Madison, WI). Briefly, H9c2 myoblasts were trypsinized, counted in a hemocytometer and ∼1×10^4^ cells seeded per well of a 96-well plate (Greiner, Kremsmünster, Austria). Cells were seeded with 300 µl DMEM. DMEM was removed from cells and 100 µl of the reconstituted assay reagent added into each well and gently mixed. Cells were subsequently incubated for ∼2 hr at room temperature and the degree of luminescence measured in white walled 96-well luminometer plates (Amersham, Buckinghamshire, UK).

**Figure 7:**
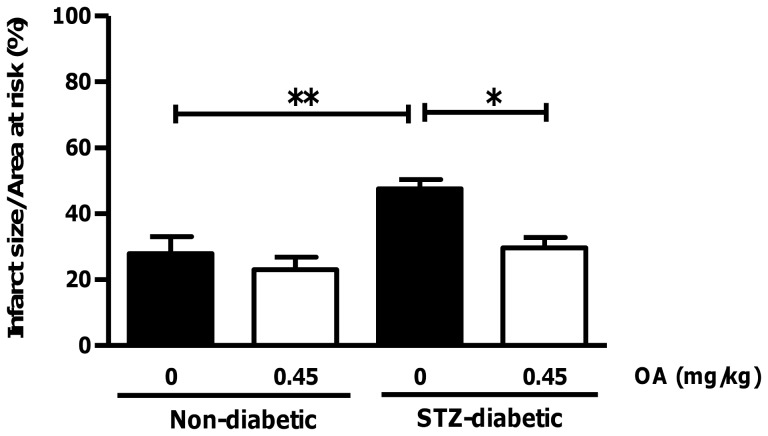
OA treatment decreases infarct size following coronary artery ligation in streptozotocin-diabetic rats. Wistar rats were injected with STZ and followed for a 1-week period. Subsequently, 0.45 mg/kg OA was injected via the penile vein within the first two min of reperfusion. Evans blue dye and TTC staining enabled visualization of viable tissue (blue), infarcted area (white) and the area at risk (red). Values are expressed as mean ± SEM (n = 6). *p<0.05, **p<0.01 vs. respective controls.

We further confirmed our apoptosis data by employing an Annexin V-FITC kit (MACS, Miltenyi Biotec, Germany) according to the manufacturer’s instructions. In brief, 100 µM OA was administered for 6 and 24 hr, respectively, as before (refer ‘Cell culture and OA treatments’ section). After the completion of our experimental protocol, cells were washed with sterile PBS (calcium free), trypsinized and centrifuged at 300 *g* for 3 min. The pellet was washed with 1 ml of 1x binding buffer per 10^6^ cells and re-centrifuged at 300 *g* for 3 min. The resulting pellet was resuspended in 100 µl of 1x binding buffer per 10^6^ cells and thereafter incubated with 10 µl of Annexin V-FITC for 15 min in the dark at room temperature. Cells were subsequently washed with 1 ml of 1x binding buffer and centrifuged at 300 *g* for 3 min. The pellet was resuspended in 500 µl of 1x binding buffer and 5 µl of PI solution immediately added, prior to analysis by flow cytometry. Viable cells (annexin V^−^PI^−^); non-viable, including late apoptotic or necrotic cells (Annexin V^+^PI^+^) or Annexin V^−^PI^+^) and apoptotic cells (Annexin V^+^PI^−^) were detected by the inding of Annexin V to externalized phosphatidylserine in conjuction with PI. Results are presented as the percentage of apoptosis normalized to control (ratio of early apoptotic, Annexin^+^/PI^−^ cells to the total population).

### Animals and Ethics Statement

All animals were treated in accordance with the Guide for the Care and use of Laboratory Animals of the National Academy of Sciences (NIH publication No. 85–23, revised 1996). Studies were performed with the approval of the Animal Ethics Committees of Stellenbosch University and the University of Cape Town (South Africa), and the United Arab Emirates University (United Arab Emirates).

**Table 1:** Body weight and blood glucose levels after 1 week of STZ injection.

	% Body weight change	Non-fasting blood glucose (mmol/L)
**Non-diabetic**	8.1±0.8	6.6±0.5
**STZ-diabetic**	−4.3±1.2	27.1±1.6***

Data are expressed as mean ± SEM, n ≥4 in each group.

*** p<0.001 vs. non-diabetic control group.

### *Ex vivo* Global Ischemia during Simulated Acute Hyperglycemia

These studies were carried out at Stellenbosch University (South Africa). Male Wistar rats weighing 180–220 gr were used throughout the study. Rats were anesthetized (pentobarbitone, 100 mg/kg *i.p*) and hearts rapidly excised and perfused in a modified Langendorff model with Krebs-Henseleit buffer equilibrated with 95% O_2_–5% CO_2_ (37°C, pH 7.4) at a constant pressure (100 cm). The Krebs-Henseleit buffer contained (in mM) 11 Glucose, 118 NaCl, 4.7 KCl, 1.2 MgSO_4_.7H_2_O, 2.5 CaCl_2_.2H_2_O, 1.2 KH_2_PO_4_, 25 NaHCO_3_. Hearts were randomly distributed into four experimental groups: 1) control (11 mM glucose), untreated; *2*) control (11 mM glucose), OA treated; 3) high glucose (33 mM glucose), untreated and 4) high glucose (33 mM glucose), OA treated (n = 9 for each group). With the high glucose perfusions we are attempting to simulate acute hyperglycemia within the clinical setting. Moreover, since *ex vivo* Langendorff perfusions are typically performed with 11 mM glucose at baseline, we are of the opinion that the 33 mM dose is representative of a 3-fold elevation of glucose levels (above normal) within the clinical setting.

The protocol was divided into two parts, i.e. perfusions a) without ischemia and b) with ischemia and reperfusion. For the non-ischemic protocol, we stabilized for 60 min whereafter 100 µM OA was added to the perfusate for an additional 20 min period. Subsequently, we returned the buffer used in the stabilization period and perfused for a further 20 min (total perfusion time: 100 min). For the ischemic protocol, 100 µM OA was added during the first 20 min of reperfusion (for OA experimental groups only; refer details below). OA was dissolved in a small volume of DMSO and less than 0.0005% (v/v) DMSO was present during perfusion experiments.

**Table 2:** Effects of OA on *in vivo* heart rate, ST height, systolic and diastolic blood pressures during early reperfusion.

	Heart rate (beats/minute)		Systolic BP (mmHg)		Diastolic BP (mmHg)	
	PI	Reperfusion	PI	Reperfusion	PI	Reperfusion
ND	404.7±16.01	411.7±8.5	130.9±8.3	125.3±9.2	81.5±14.1	107.6±7.4
ND + OA	411.6±12.4	399.7±15.1	113.6±13.9	88.6±9.5*	87.5±2.5	62.9±9.1*
STZ	394.3±35.0	429.6±20.4	127.4±8.9	118.4±8.9	63.3±4.7	100.8±3.6
STZ + OA	386.3±14.7	391.0±17.8	117.4±5.8	99.0±5.0*	53.7±4.5	65.0±8.0*

ND (non-diabetic); STZ (STZ-diabetic); BP (blood pressure); PI (pre-ischemic). Data are expressed as mean ± SEM, n ≥4 in each group).

* p<0.05 vs. respective control.

During perfusion, a latex balloon attached to a pressure transducer (Stratham MLT 0380/D, AD Instruments Inc., Bella Vista, NSW, Australia) compatible with the PowerLab System ML410/W (AD Instruments Inc., Bella Vista, NSW, Australia), was inserted into the left ventricle and inflated to produce a diastolic pressure of 4–12 mm Hg. The protocol had a 60 min stabilization period, 20 min of global ischemia, followed by reperfusion for a further 60 min. Contractile parameters assessed included heart rate (HR), left ventricular developed pressure (LVDP), and rate-pressure product (RPP; RPP = HR×LVDP). Left ventricular tissues were collected at 4 time points, i.e. a) within the first two min after ischemia, b) 20 and c) 40 min after ischemia, respectively, and d) also after 60 min of reperfusion. Collected tissues were freeze-clamped in liquid nitrogen and stored at −80°C for further analysis.

### *Ex vivo* Regional Ischemia during Simulated Acute Hyperglycemia

These studies were carried out at Stellenbosch University (South Africa). To further strengthen our Langendorff perfusion data we also evaluated the effects of OA by infarct size determination. This was performed as described before but with slight modifications [27], i.e. we employed regional ischemia with a reperfusion time of 2 hr. Here a 3/0 silk suture was placed on the proximal portion of the left anterior descending coronary artery and passing the ends through a plastic tube. For induction of regional ischemia, the ends were tightened by pressing the plastic tube against the surface of the heart (above the artery) for 20 min. The snare was released during the reperfusion period. The efficacy of ischemia was confirmed by regional cyanosis and a substantial decrease in coronary flow.

**Figure 8:**
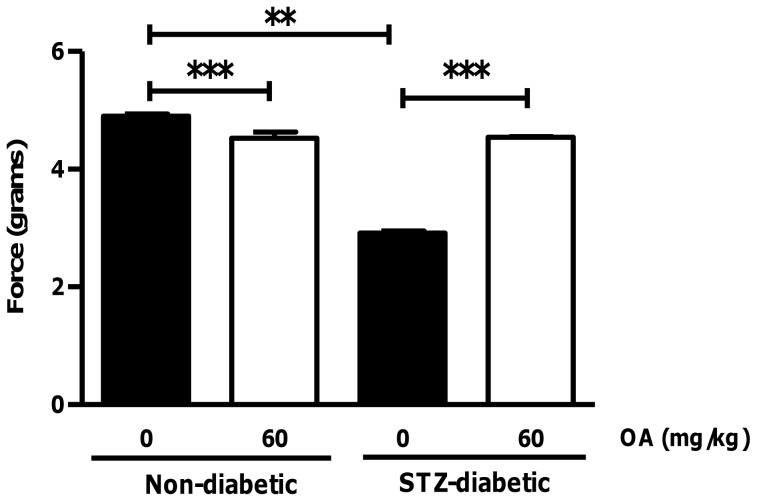
Long-term OA treatment improves cardiac function in STZ-diabetic rats. Sprague-Dawley rats were injected with STZ and followed for a 2-week period ± daily OA treatment. Subsequently, isolated hearts from STZ-diabetic and matched controls were perfused and action potentials recorded via a force transducer. Values are expressed as mean ± SEM (n = 6). **p<0.05 vs. non-diabetic control and ***p<0.01 vs. respective controls.

### Determination of Infarct Size

After completion of each regional ischemia-reperfusion experiment the snare was re-tightened and 2.5% Evans Blue dye (in Krebs buffer) was perfused through the hearts for infarct development. Hearts were subsequently removed from the Langendorff apparatus, blotted dry, suspended within 50 ml plastic tubes (using suture) and frozen at −20°C for 3 days. Thereafter, frozen hearts were sliced into 2 mm transverse sections and incubated with 1% 2,3,5-triphenyl tetrazolium chloride (TTC) in phosphate-buffered saline for 20 min at 37°C to identify non-infarcted (stained) from infarcted (non-stained) tissues. Slices were then fixed in 10% formalin for 24 hr at room temperature before being placed between glass plates for scanning (both sides). The infarct area (IA) size and the area at risk (AAR) were calculated using Image J software (v1.46p, National Institutes of Health, USA). Values of tissue slices were added together in order to obtain the total IA and AAR for each heart analyzed. We expressed the infarct size as the ratio of IA versus the AAR (%IA/AAR).

**Figure 9:**
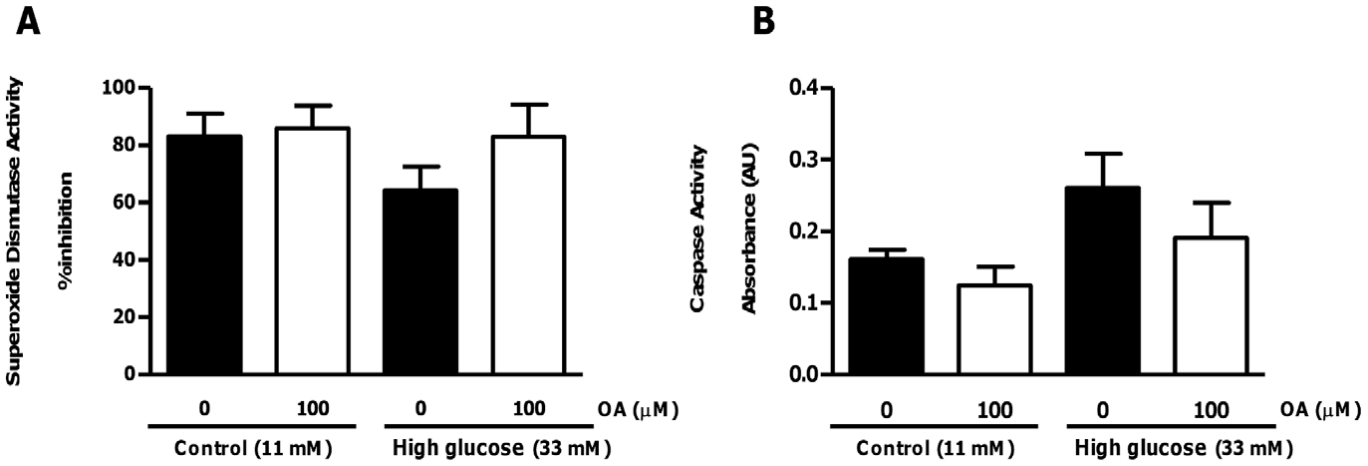
OA treatment does not affect pre-ischemic superoxide dismutase and caspase activities. Isolated rat hearts were perfused under simulated hyperglycemic conditions (33 mM glucose) vs. controls (11 mM glucose) ±100 µM OA treatment. We initially perfused for 60 min, whereafter OA was added for a further 20 min. Subsequently, the buffer initially used was returned and hearts perfused for an additional 20 min. (A) Superoxide dismutase activity (% inhibition) in response to high glucose vs. control ± OA treatment; (B) Caspase activity. Values are expressed as mean ± SEM (n = 6).

### *In vivo* Regional Ischemia during Chronic Hyperglycemia (Streptozotocin-treated Rats)

We next tested the effects of OA on diabetic rats (chronic hyperglycemia) subjected to an ischemic insult. These experiments were completed at the University of Cape Town (South Africa). Hyperglycemia was induced in Wistar rats as previously described [21], [25]. In brief, male Wistar rats weighing 250–300 gr were injected (intraperitoneally) with a single dose of streptozotocin (STZ) (60 mg/kg dissolved in freshly prepared 0.1 M citrate buffer [pH 6.2]; Sigma-Aldrich, St Louis MO). Control rats were injected with the vehicle (citrate buffer). Blood glucose concentrations of ≥20 mmol/l after 1 week were considered as a stable diabetic state before experimental procedures. We also determined body weights and non-fasting blood glucose levels before STZ induction and after the one week of diabetes induction.

**Figure 10:**
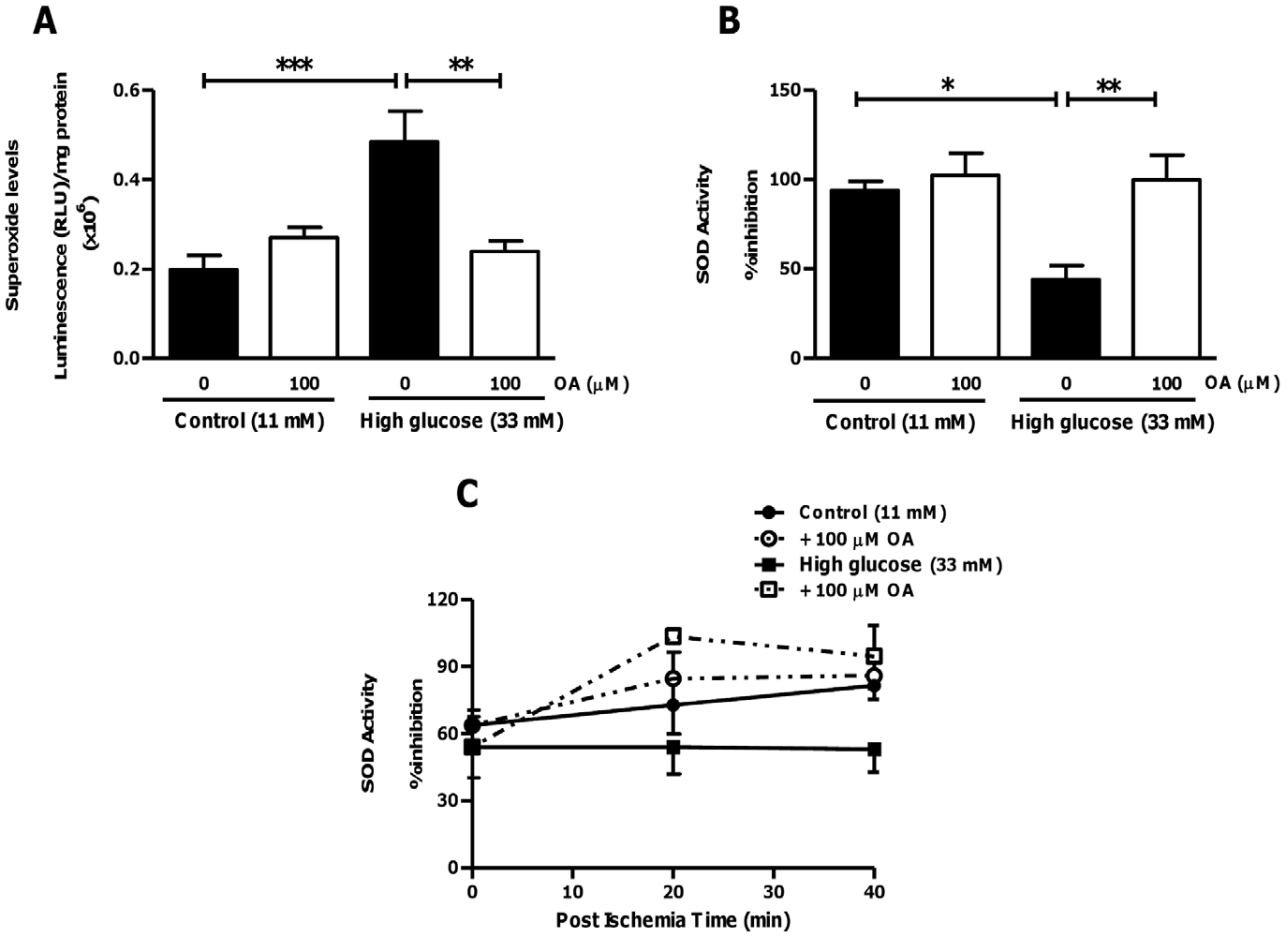
Anti-oxidant effects of OA in hearts subjected to ischemia-reperfusion under high glucose perfusion conditions. Isolated rat hearts were perfused under high glucose conditions vs. controls and subjected to ischemia-reperfusion. For OA treatments groups, 100 µM OA was added during the first 20 min of reperfusion. (A) Superoxide levels under high glucose conditions vs. control ± OA treatment; (B) Superoxide dismutase activity (% inhibition) in response to high glucose vs. control ± OA treatment; (C) Time course for SOD activity following ischemic insult. Values are expressed as mean ± SEM (n = 9). *p<0.05, **p<0.01, ***p<0.001 vs. respective controls.

For the *in vivo* coronary artery ligation experiments, rats were divided into control (citrate-treated) and diabetic (STZ-treated) groups. Each group was subjected to coronary artery ligations ± OA treatment (0.45 mg/kg i.v) (as described in detail below). The OA was dissolved in <0.001% DMSO and deionized water; freshly done for each treatment period.

Ligation experiments were performed one week after STZ intraperitoneal injection and confirmation of a stable diabetic state. Rats were anesthetized with sodium pentobarbital (60 mg/kg i.p.), intubated, and thereafter ventilated with room air (2.5 ml/stroke) at a rate of 75 strokes per min via a rodent ventilator (Model 681, Harvard Apparatus, USA). Body temperature was monitored by a rectal temperature probe and a constant temperature was maintained throughout the surgical procedure by placing rats on a custom-made heating block. The depth of anesthesia was checked by assessing the pedal withdrawal reflex and by monitoring heart rate. Maintenance doses of anesthetic (6 mg/kg i.p) were administered as required. Lead II electrocardiogram (ECG) was recorded via an Animal Bio Amplifier (ML136, ADInstruments, Castle Hill, New South Wales, Australia). Carotid arterial blood pressure was recorded via a custom-made cannula attached to a pressure transducer (MLT0670, ADInstruments, Castle Hill, New South Wales, Australia). Since formation of clots around intra-arterial cannulae poses a potential risk for arterial thrombosis, heparin (1000 IU/kg i.p) was injected concurrently with anesthetic [28], [29].

**Figure 11:**
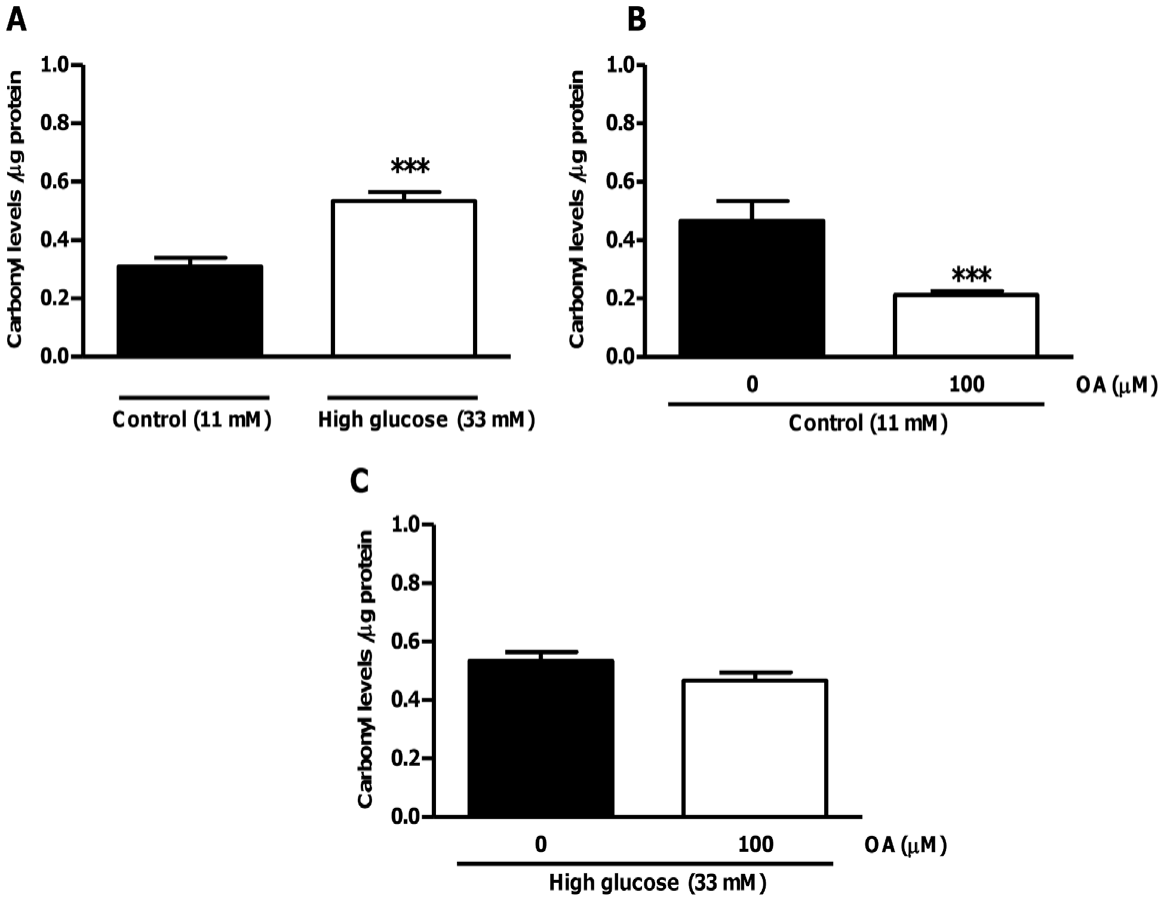
OA treatment decreases carbonylation levels in hearts subjected to ischemia-reperfusion under high glucose conditions. Isolated rat hearts were perfused under high glucose conditions vs. controls and subjected to ischemia-reperfusion. For OA treatments groups, 100 µM OA was added during the first 20 min of reperfusion. (A) Degree of carbonylation under high glucose conditions vs. control; (B) OA treatment at baseline (11 mM glucose) and (C) under high glucose conditions (33 mM). Values are expressed as mean ± SEM (n = 9). ***p<0.001 vs. respective controls.

A left thoracotomy was performed through the 4^th^ intercostal space and the left lung collapsed using a damp swab. The left anterior descending coronary artery was thereafter ligated as previously described [30]. A 6–0 silk suture was placed around the left anterior descending coronary artery and its ends passed through a plastic tube. For induction of regional ischemia the ends of the suture were used as a snare to occlude the artery by applying it gently onto the ventricular surface for 30 min. The efficacy of ischemia was confirmed by regional cyanosis and ECG changes. We employed S-T elevation (ECG) to confirm coronary artery ligation. The snare was released during reperfusion.

Rat hearts were subjected to 30 min ischemia followed by 2 hr of reperfusion. The penile vein was cannulated for OA administration, while the vehicle solution was administered to control animals. For the OA rats, a bolus dose of 0.45 mg/kg i.v [21] was injected immediately on reperfusion (within 1 min of releasing the snare) and therafter reperfused for 2 hr as before. After the reperfusion period, the heart was flushed with saline and the coronary artery was re-occluded with the suture that had been left in place. The heart was then stained with 2.5% Evans blue to reveal the AAR. TTC staining and infarct sizes were determined as described for the *ex vivo* model.

### Effects of Long-term OA Treatment on Heart Function in Streptozotocin-treated Rats (Chronic Hyperglycemia)

We also ascertained the effects of chronic OA treatment within the diabetic context and its effects on cardiac function. These studies were completed at the United Arab Emirates University (Al-Ain, United Arab Emirates). Here diabetes was induced in male Sprague-Dawley rats by a single intraperitoneal injection of STZ (60 mg/kg body weight) dissolved in citrate buffer. For OA-treated rats daily oral gavage was performed with a dose of 60 mg/kg for the entire 2-week period. The OA was made up freshly on each day and dissolved in DMSO and water. At the end of the two week period, we determined body weights and fasting blood glucose levels for all experiments.

**Figure 12:**
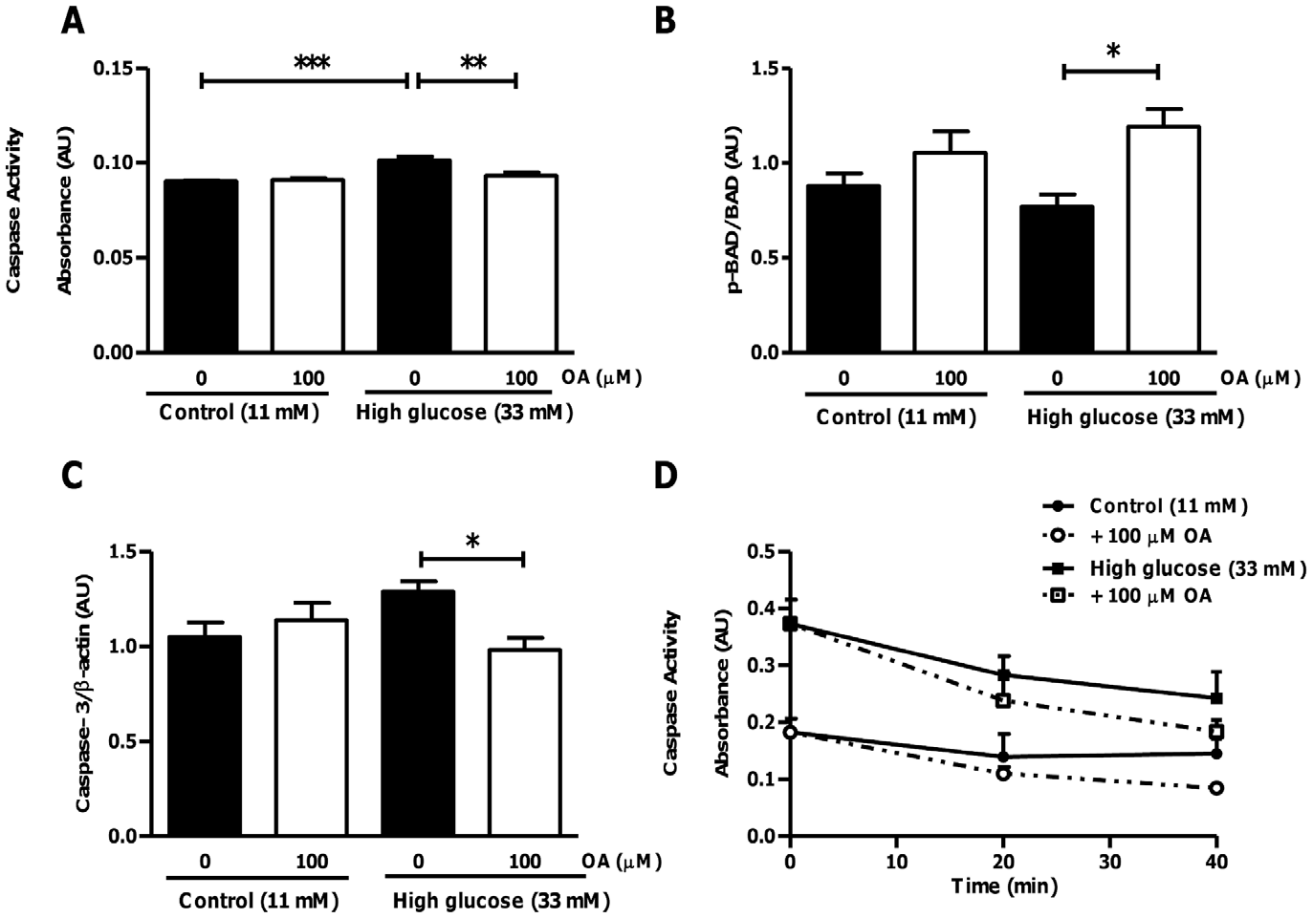
Anti-apoptotic effects of OA in hearts subjected to ischemia-reperfusion under high glucose conditions. Isolated rat hearts were perfused under high glucose conditions vs. controls and subjected to ischemia-reperfusion. For OA treatments groups, 100 µM OA was added during the first 20 min of reperfusion. (A) Caspase activity; (B) p-BAD/BAD peptide levels; (C) Caspase-3 peptide levels; (D) Time course of myocardial apoptosis following the ischemic insult. Values are expressed as mean ± SEM (n = 9). *p<0.05, **p<0.01, ***p<0.001 vs. respective controls.

Following STZ treatment, rats were killed by decapitation and hearts rapidly removed, mounted in Langendorff mode and perfused at a constant flow of 8 ml (g heart)^−1^ min^−1^ at physiological temperature (36–37°C) with Tyrode solution containing: 140 mM NaCl; 5 mM KCl; 1 mM MgCl_2_; 10 mM glucose; 5 mM HEPES; and 1.8 mM CaCl_2_; adjusted to pH 7.4 with NaOH and continuously bubbled with oxygen. When the heart rate stabilized, extracellular action potentials were recorded from the left ventricle in spontaneously beating hearts with a purpose-built extracellular suction electrode as previously described [31]. Here a clip is fixed at the apex of the hanging heart and a thread tied to the clip. The thread was guided through pulleys and tied to the force transducer that was connected to the Power Lab system. Signals from the electrode were collected at 400 Hz, amplified (ML136 Bioamp, ADInstruments, Castle Hill, New South Wales, Australia) and conveyed via a Powerlab (PL410, ADInstruments, Castle Hill, New South Wales, Australia) to a personal computer. Heart functional data are expressed as force generated (grams).

**Figure 13:**
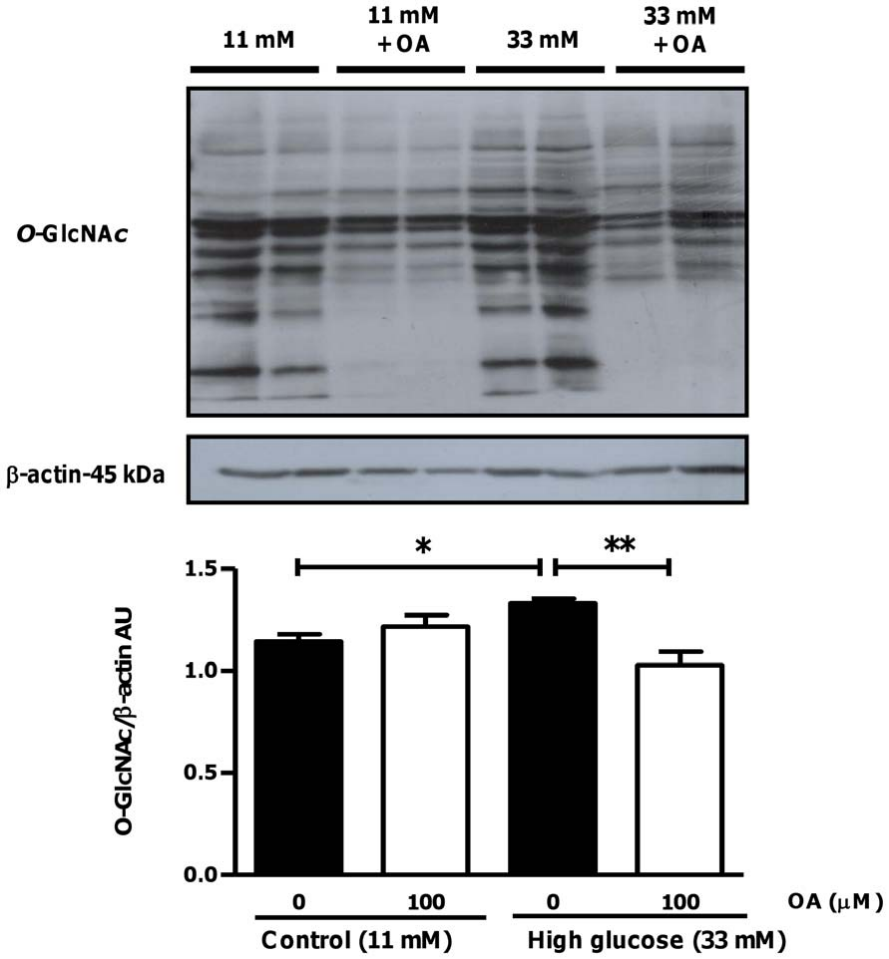
OA treatment attenuates *O*-GlcNAcylation in hearts subjected to ischemia-reperfusion under high glucose conditions. Isolated rat hearts were perfused under high glucose conditions vs. controls and subjected to ischemia-reperfusion ± OA treatment during reperfusion. Western blot analysis for overall *O*-GlcNAcylation is shown with β–actin as loading control. Densitometric analysis for *O*-GlcNAcylation is displayed below gel image (normalized to corresponding β–actin values). Values are expressed as mean ± SEM (n = 6). *p<0.05, **p<0.01 vs. respective controls.

### Western Blot Analysis

Protein isolation was performed as previously described [13]. Briefly, collected heart tissues were homogenized with modified RIPA buffer, the supernatant centrifuged twice at 4, 300 *g* for 10 min at 4°C then stored at −80°C until further use. Protein expression was determined by Western blotting as described before by us [13], [14] for the following antibodies: BAD, phosphorylated-BAD (Ser 136), caspase-3 (Cell Signaling, MA, USA), and *O*-GlcNAc (HBP marker; CTD110.6, Santa Cruz Biotechnology Inc.). We employed β–actin (Cell Signaling, MA, USA) as a loading control.

### Measurement of Superoxide Dismutase (SOD) Activity

We assessed the total SOD activity (cytosolic and mitochondrial components) as detailed in the instructions of a commercially obtained kit (Biovision K 335–100, Mountain View, CA 94043 USA). The assay depends on utilizing a highly water-soluble tetrazolium salt, WST-1 (2-(4-iodophenyl)-3-(4-nitrophenyl)-5-(2,4-disulfo-phenyl)-2H-tetrazolium, monosodium salt), which produces a water-soluble formazan dye upon reduction with a superoxide anion. The rate of WST-1 reduction by superoxide anion is linearly related to the xanthine oxidase activity and is inhibited by SOD. Formazan levels can be measured by absorption with a spectrophotometer at 450 nm. Briefly, collected heart tissues were homogenized with modified ice cold RIPA buffer, the supernatant centrifuged twice at 4, 300 *g* for 10 min at 4°C. The samples were incubated with the enzyme and WST working solutions at 37°C for 20 min in a 96 well-microtiter-plate (Corning, New York, USA) in an orbital shaker incubator. Absorbance was read at 450 nm with a microplate reader (EL 800 KC Junior Universal Microplate reader, Bio-Tek Instruments Inc, Vermont, USA). SOD activity was calculated according to the following formula: % inhibition = (A_control_–A_sample_)/A_control_×100.

**Figure 14:**
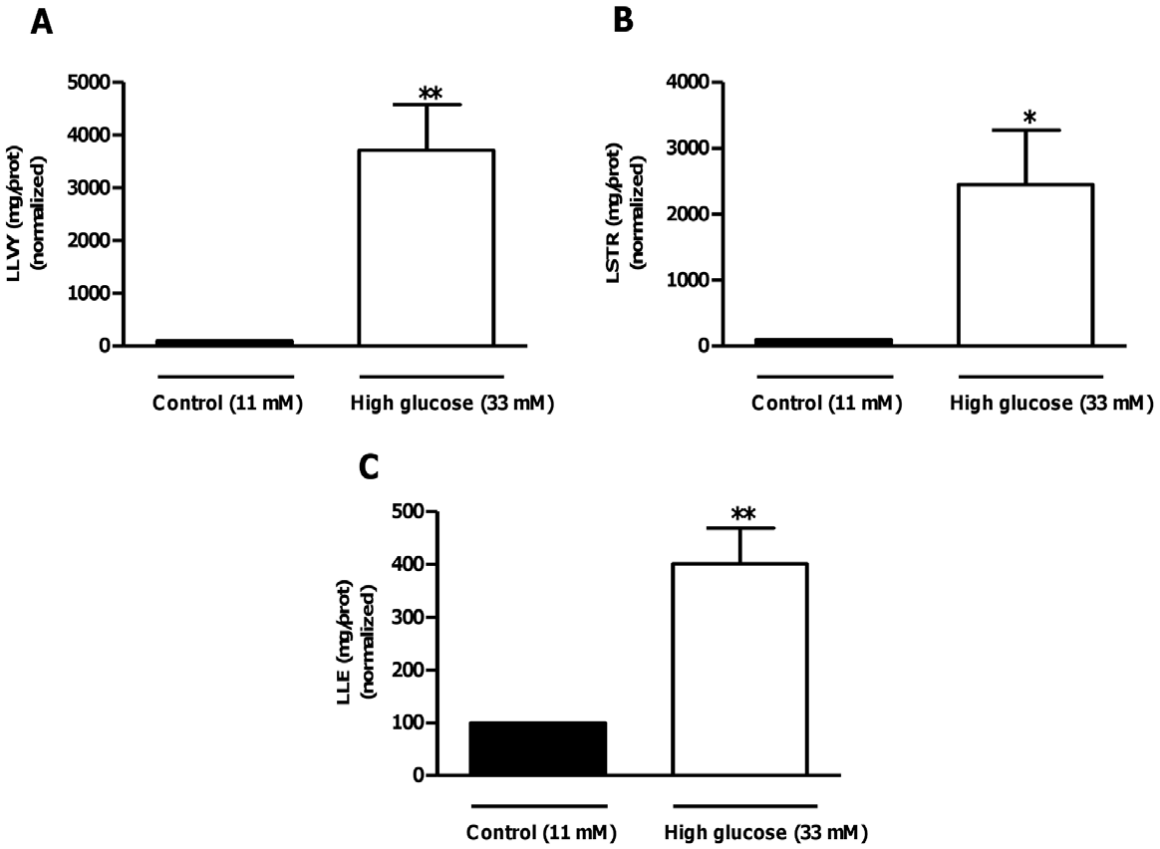
Increased proteasomal activity in hearts subjected to ischemia-reperfusion under high glucose conditions. Isolated rat hearts were perfused under high glucose conditions vs. controls and subjected to ischemia-reperfusion. A) Trypsin-like proteasomal, (B) chymotrypsin-like, and (C) caspase-like activities after 60 min of reperfusion under high glucose conditions (33 mM) vs. control (11 mM). Values are expressed as mean ± SEM (n = 9). *p<0.05, **p<0.01 vs. respective controls.

### Myocardial Superoxide Levels

The heart tissue was pulverized and homogenized in 100 volumes of perchloric acid (10% v/v) and centrifuged for 20 min at 13, 000 *g* [32]. Protein-free supernatant (0.1 ml) was subsequently incubated with 0.25 mM lucigenin (Sigma-Aldrich, St. Louis, MO) at room temperature for 5 min in the dark and chemiluminescence measured in a white-walled luminometer 96 well-microtiter plate (Corning, New York, USA). Superoxide levels were expressed as chemiluminescence (RLU) per mg tissue.

### Isolation of Proteins for Carbonylation and Proteasome Activity Experiments

Heart tissues were cut into small slices and homogenized in 1 ml of Tris-HCl buffer (pH 7.4) using an IKA Ultra Turrax T25 homogenizer (IKA Labortechnik, Staufen, Germany) and incubated on ice for 10 min before centrifugation at 9, 000 *g* for 15 min to remove cell debris. The supernatant was used for protein quantification using the BCA assay.

### ELISA Carbonyl Protocol

Protein carbonyls are formed by a variety of oxidative mechanisms and are sensitive indices of oxidative injury. Protein carbonylation was determined by the carbonyl ELISA assay developed in the GEICO laboratory (Université de La Réunion, Saint Denis de La Réunion, France) based on recognition of protein-bound DNPH in carbonylated proteins with an anti-DNP antibody [33].

**Figure 15:**
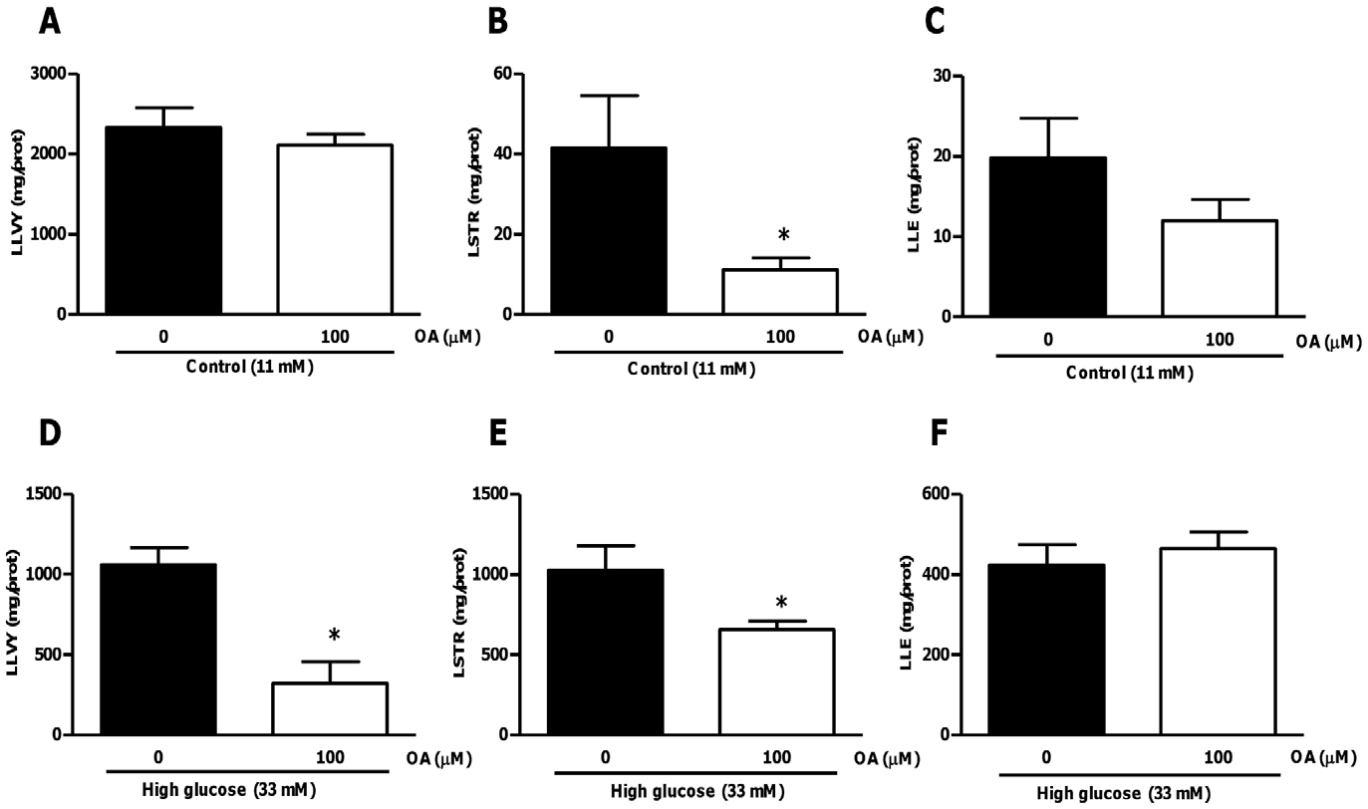
OA attenuates high glucose-induced proteasomal activity following ischemia-reperfusion. Isolated rat hearts were perfused under high glucose conditions vs. controls and subjected to ischemia-reperfusion. For OA treatments groups, 100 µM OA was added during the first 20 min of reperfusion. (A) Trypsin-like proteasomal, (B) chymotrypsin-like, and (C) caspase-like activities after 60 min of reperfusion at baseline (11 mM glucose). (D) Trypsin-like proteasomal, (E) chymotrypsin-like, and (F) caspase-like activities after 60 min of reperfusion under high glucose conditions (33 mM glucose). Values are expressed as mean ± SEM (n = 9). *p<0.05 vs. respective controls.

Here 5 µl of protein from tissue lysates (0.2–0.6 µg) was denatured by adding 10 µl 12% SDS solution. Subsequently, proteins were derivatized to DNP hydrazone with 10 µl of DNPH solution (10 mM in 6 M guanidine hydrochloride, 0.5 M potassium phosphate buffer, pH 2.5). DNPH is a chemical compound that specifically reacts and binds to carbonylated proteins. Samples were incubated at room temperature for 30 min and the reaction was neutralized and diluted in coating buffer (10 mM sodium carbonate buffer, pH 9.6) to yield a final protein concentration of 0.2–0.6 ng/µl.

Diluted samples were added to wells of a Nunc Immuno Plate Maxisorp (Dutscher, Brumath, France) and incubated at 37°C for 3 hr, and thereafter washed 5x with PBS/Tween (0.1%) between each of the following steps: blocking the wells with 1% BSA in PBS/Tween (0.1%) overnight at 4°C; incubation with anti-DNP antibody (Sigma-Aldrich, St Louis, MO) (1∶2000 dilution in PBS/Tween [0.1%]/BSA [1%]) at 37°C for 3 hr; incubation with horse radish peroxidase-conjugated polyclonal anti-rabbit immunoglobulin (GE Healthcare, Mannheim, Germany) (1∶4000 dilution in PBS/Tween [0.1%]/BSA [1%]) for 1 hr at 37°C; addition of 100 µl of TMB substrate solution and incubation for 10 min before stopping the coloration with 100 µl of 2 M sulphuric acid. Absorbances were read at 490 nm against the blank (DNP reagent in coating buffer without protein) with a Fluostar microplate reader (BMG Labtech, Ortenberg, Germany). Results are expressed as percentage of absorbance compared to control cells (treatment of samples with 11 mM glucose) after normalization with protein concentrations.

### Proteasome Activity Measurements

Chymotrypsin-like, trypsin-like, and caspase-like activities of the proteasome were assayed using fluorogenic peptides (Sigma-Aldrich, St Louis, MO): Suc-Leu-Leu-Val-Tyr-7-amido-4-methylcoumarin (LLVY-MCA at 25 µM), N-t-Boc-Leu-Ser-Thr-Arg-7-amido-4-methylcoumarin (LSTR-MCA at 40 µM) and N-Cbz-Leu-Leu-Glu-b-naphthylamide (LLE-NA at 150 µM), respectively [34]. Assays were performed with ∼50 µg of protein lysate (in 25 mM Tris–HCl [pH 7.5]) and the appropriate substrate that were incubated together for 0–30 min at 37°C. Aminomethylcoumarin and β-naphthylamine fluorescence were measured at excitation/emission wavelengths of 350/440 and 333/410 nm, respectively, using a Fluostar fluorometric microplate reader (BMG Labtech, Ortenberg, Germany). Peptidase activities were measured in the absence/presence of 20 µM of the proteasome inhibitor, MG132 (N-Cbz-Leu-Leu-leucinal), and the difference between the two values was attributed to proteasome activity. Data were normalized to protein concentrations.

### Statistical Analysis

Data are presented as mean ± standard error of mean (SEM). Statistical analysis was performed by the Mann Whitney t-test, or one way analysis of variance (ANOVA) and Tukey –Kramer (GraphPad Prism). Values were considered significant when p<0.05.

## Results

### Isolation of OA from Clove Extract

Evaluation of one-dimensional ^1^H- and ^13^C-NMR spectra of the isolated OA compound confirmed the presence of the 48 hydrogen and the 30 carbon atoms present in the molecule (data not shown). For the determination of complex components within the molecule, two- dimensional ^1^H- and ^13^C-NMR spectra was conducted and confirmed the chemical structure of OA (data not shown).

### Effects of OA Treatment on ROS Levels and Apoptosis in Heart Cells

We employed both fluorescence microscopy and flow cytometric analysis to evaluate whether OA acts as an anti-oxidant under *in vitro* simulated chronic hyperglycemic conditions. Our data show significantly increased ROS levels in heart cells that were cultured under high glucose conditions (Figure 1). However, OA treatment (acute and chronic) significantly decreased myocardial ROS levels under control and high glucose conditions. Here even the lower OA dose (20 µM) blunted the increase in ROS levels under high glucose culturing conditions (p<0.05 vs. untreated high glucose cells) (Figure 1).

To examine whether OA exhibits anti-apoptotic effects, H9c2 cells were treated with 20 µM and 50 µM OA, respectively, for 24 hr. Our data revealed increased caspase-3 activity in response to high glucose culturing conditions (p<0.05 vs. 5.5 mM glucose group) (Figure 2). The low glucose-treated cells also exhibited a dose-dependent reduction in caspase-3 enzymatic activity, i.e. by 28.4±2.2% and by 43.7±1.5%, respectively, for the 20 µM and 50 µM OA doses (p<0.001 vs. 5.5 mM glucose group). Likewise, high glucose-treated cells displayed a decrease, i.e. by 19.9±0.8% and by 43.4±3.4%, respectively, for the 20 µM and 50 µM OA doses (p<0.001 vs. 33 mM glucose control) (Figure 2). Our results at the 6 hr time point revealed similar findings (data not shown). In support, flow cytometric analysis (Annexin V/FITC PI) showed decreased apoptosis in H9c2 cells exposed to high glucose and treated with 100 µM OA for 6 hr (p<0.01) (Figure 3). After 24 hr the decrease was observed under both low (p<0.001 vs. control) and high glucose (p<0.001 vs. control; p<0.001 vs. high glucose without OA treatment) culturing conditions (Figure 3).

### Evaluation of *ex vivo* Heart Function during Global Ischemia-reperfusion (Simulated Acute Hyperglycemia)

The functional data for both low and high glucose perfusions (without ischemia) showed no significant differences with OA treatment (Figure 4). However, perfusion data with global ischemia and reperfusion show that LVDP recovery for hearts perfused with high glucose was markedly lower compared to the control group (p<0.01 vs. 11 mM control), ranging between 12.7±3.2% and 23.1±0.9% of the baseline pre-ischemic values during reperfusion (Figure 5). By contrast, the control group’s functional recovery during reperfusion ranged from ∼28 to 50% of the baseline pre-ischemic value. Our perfusion data revealed that OA treatment markedly improved functional recovery of high glucose perfused hearts during reperfusion (p<0.001 vs. untreated high glucose), i.e. to 62±8% of the baseline pre-ischemic value (Figure 5B). In agreement, OA treatment enhanced dP/dt*_max_* (data not shown) and RPP for the high glucose perfused group while it resulted in no significant effects for baseline treated hearts (Figure 5). Moreover, the regional ischemia-reperfusion experiments show that OA treatment of high glucose perfused hearts decreased the infarct size from 59.6±3.1% to 38.7±1.6% (p<0.01 vs. high glucose untreated) (Figure 6). We also performed additional experiments with 11 mM and 33 mM mannitol and found no significant effects on functional recovery of hearts following global ischemia-reperfusion, thus ruling out any osmotic effects (data not shown).

### *In vivo* Coronary Artery Ligations in Streptozotocin-treated Rats (Chronic Hyperglycemia)

We next evaluated the cardioprotective effects of OA treatment within the *in vivo* context, i.e. by employing coronary artery ligations in STZ-treated hyperglycemic rats. Our data revealed markedly elevated blood glucose levels in STZ-diabetic rats compared to matched controls (p<0.001) (Table 1). This was associated with decreased weight gain in the STZ-diabetic rats. In agreement with our *ex vivo* perfusion data, we found that infarct size was significantly increased for the STZ-treated rats versus controls (47.5±2.9% vs. 27.9±5.2%) following 30 min coronary artery ligation (Figure 7). Moreover, this effect was blunted by OA treatment. There were no differences in the area at risk amongst all the groups (data not shown). OA administration also decreased systolic and diastolic blood pressures in STZ-diabetic rats vs. untreated STZ-diabetic controls during the early reperfusion period, i.e. within the first twenty minutes of reperfusion (Table 2). There were no significant differences on heart rate and systolic/diastolic blood pressures before regional ischemia for all the groups (Table 2).

### Effects of Long-term OA Treatment on Heart Function in Streptozotocin-treated Rats (Chronic Hyperglycemia)

To assess the effects of long-term OA treatment on heart function, rats were injected with STZ and followed for 2 weeks ± daily OA treatment. STZ treatment markedly increased fasting blood glucose (16.3±0.8 mmol/L) versus matched controls (5.4±0.3 mmol/L) (p<0.01). Our data show that the force generated by STZ-diabetic rat hearts was significantly lower compared to non-diabetic controls (p<0.01) (Figure 8). However, chronic OA treatment for two weeks significantly improved cardiac function in STZ-diabetic rats.

### Effects of OA Treatment on *ex vivo* Myocardial ROS Levels and Apoptosis

To confirm our *in vitro* data, we next evaluated whether OA exhibits anti-oxidant and anti-apoptotic properties within an *ex vivo* context. Under pre-ischemic conditions OA treatment did not significantly affect myocardial SOD activity or apoptosis (Figure 9). However, following ischemia OA administration blunted high glucose-induced myocardial superoxide levels and concomitantly upregulated SOD activity (Figure 10A and 10B). To gain further insight into temporal effects, we also performed analyses immediately after ischemia and for several time points thereafter. These data show that OA exerts its main anti-oxidant effects within the first 20 minutes following ischemia, whereafter it is sustained (Figure 10C). We largely confirmed these data by evaluating the degree of protein carbonylation as an additional marker of oxidative stress (Figure 11). However, the OA-induced decrease in protein carbonylation under high glucose conditions did not reach statistical significance (p = 0.076 vs. untreated high glucose) (Figure 11C).

We next assessed anti-apoptotic effects of OA in *ex vivo* perfused heart tissues. Myocardial caspase activity levels were increased under high glucose perfusion conditions (p<0.001 vs. untreated control). This effect was attenuated by OA treatment (p<0.01 vs. untreated high glucose) (Figure 12A). In agreement, OA treatment significantly increased cardiac p-BAD/BAD and decreased caspase-3 peptide levels under high glucose perfusion conditions (Figure 12B and 12C). We also investigated the temporal nature of myocardial apoptosis following ischemia. Our data show that anti-apoptotic effects emerge at the 40 minute time point, suggesting that these effects occur as a result of the earlier upstream reduction of oxidative stress (Figure 12D).

To evaluate whether OA mediates its anti-apoptotic effects via HBP modulation, we next determined the overall degree of *O*-GlcNAcylation in our experimental system. Our data show increased *O*-GlcNAcylation in response to high glucose exposure (p<0.05 vs. untreated high glucose) that was significantly decreased by OA treatment (Figure 13).

### Evaluating the Effects of OA Treatment on Myocardial Proteasomal Activity in Hearts Subjected to Ischemia-reperfusion under High Glucose Conditions

We initially evaluated post-ischemic proteasomal activities under high glucose perfusion conditions. Here we found that all proteasomal activities were markedly induced in high glucose perfused hearts following ischemia-reperfusion (Figure 14). For 11 mM glucose perfusions, OA treatment decreased chymotrypsin-like proteasomal activity compared to the untreated control (p<0.05), but did not result in any significant effects on trypsin-like and caspase-like proteasomal activities (Figure 15A, B, C). Furthermore, OA treatment significantly diminished trypsin-like and chymotrypsin-like proteasomal activities in high glucose exposed rat hearts (Figure 15D and 15E). However, it had no effect on caspase-like activity in these hearts (Figure 15F).

## Discussion

The damaging alliance between hyperglycemia and myocardial infarctions necessitates the development of novel therapeutic interventions that offer cardioprotection within this context. Moreover, stress-induced hyperglycemia in non-diabetic patients suffering an acute myocardial infarction also results in damaging outcomes, e.g. increased in-hospital mortality [7], [8]. For the current study we explored whether OA acts as a novel cardioprotective factor in response to ischemia-reperfusion under hyperglycemic conditions. We tested our hypothesis by employing cell culture studies, *ex vivo and in vivo* rat heart perfusion models, and long-term OA treatment of diabetic rats as experimental systems. The main findings of this study are that OA treatment results in cardioprotection for rat hearts by attenuating hyperglycemia-induced oxidative stress, apoptosis, and proteasomal activity following ischemia-reperfusion.

We initially investigated our hypothesis by exposing isolated rat hearts to ischemia-reperfusion and found a marked decline in contractile function under high glucose perfusion conditions (simulated acute hyperglycemia). However, OA treatment resulted in a striking increase in functional recovery and demonstrates, for the first time as far as we are aware, that it triggers cardioprotection in high glucose perfused rat hearts. Our infarct size data further corroborated these findings. Moreover, OA administration following *in vivo* coronary artery ligations in hyperglycemic rats also decreased infarct sizes in the STZ-diabetic rats. Although OA-mediated cardioprotection was previously reported following ischemia-reperfusion, such studies were performed on hearts isolated from rats that were pre-treated with OA for several days [22], [35]. Furthermore, these experiments were completed under normoglycemic perfusion conditions. We also performed experiments for long-term OA treatment and found that it restored heart function in isolated rat heart perfusions. Collectively, our data therefore demonstrate that OA treatment initiated *after* an ischemic insult elicited cardioprotection, thus meaning that our findings may offer therapeutic value to actual clinical settings (versus pre-treatments).

To gain further insight into underlying mechanisms responsible for OA-mediated cardioprotection, we next evaluated whether it displays anti-oxidant and anti-apoptotic properties in our experimental systems. Here we found that OA diminished high glucose-induced myocardial oxidative stress and apoptosis in both our *in vitro* and *ex vivo* experimental models. Moreover, our *ex vivo* data show significantly elevated superoxide levels together with decreased SOD activity in simulated acute hyperglycemic hearts following an ischemic insult. How does OA exert its anti-oxidant effects in hyperglycemic hearts exposed to ischemia-reperfusion? Although the precise mechanisms of OA-mediated anti-oxidant effects are unclear, earlier data support a direct scavenging role, i.e. by decreasing superoxide and hydrogen peroxide levels together with reduced lipid peroxidation [20], [36]. Here OA (a lipophilic compound) is proposed to intercalate into the lipid matrix and stabilize myocardial cell membranes under stressful conditions [37]. In addition, OA contains a single phenolic hydroxyl group that is likely to be the bioactive group for scavenging free radicals [38]. Our time course studies suggest that this likely occurs quite rapidly after an ischemic insult, peaking at about 20 minutes and thereafter sustained up to at least 60 minutes post-ischemia.

Previous studies established that OA treatment also results in transcriptional effects that increase expression of several anti-oxidant defense genes, including α-tocopherol, glutathione peroxidase, catalase, thioredoxin peroxidase and superoxide dismutase [35], [38]–[42]. Higher gene expression in this case is probably mediated by MAP kinases (JNK and ERK) that may regulate target transcriptional modulators [39]. The basic leucine zipper transcription factor, nuclear factor erythroid 2 p45-related factor 2 (Nrf2), is strongly implicated in this process [39]. Nrf2 plays a key role in protecting cells from oxidative stress by binding anti-oxidant-response elements (ARE) of gene promoters to induce expression of numerous target genes, e.g. glutathione peroxidase, heme oxygenase, and thioredoxin [43]–[45]. Earlier work determined that OA upregulated Nrf2 expression in hepatocytes, with MAPK activation implicated in this process [39]. Furthermore, Ichikawa et al. [46] found that dh404 (novel OA derivative) increased Nrf2 translocation to the nucleus to enhance Nrf2-driven transcription and suppress oxidative stress in H9c2 cardiomyoblasts. Collectively these data show that OA acts as an anti-oxidant and that its effects are mediated both by a direct scavenging role and also by induction of various anti-oxidant defense genes.

We propose that the improved functional effects detected with OA treatment stems in part from its anti-apoptotic effects observed in both our experimental models, that likely occur downstream of elevated oxidative stress. There are several ways how this may happen. Firstly, rat hearts exposed to high glucose displayed increased superoxide and nitric oxide generation that favors the production of peroxynitrite and nitrotyrosine with damaging consequences [47]. For example, others found that peroxynitrite inhibits the mitochondrial respiratory chain [48], [49] and triggers myocardial apoptosis [50]. Our time course studies support the concept that myocardial apoptosis occurs downstream of the initial post-ischemic oxidative stress burst. Here we found that myocardial apoptosis in high glucose perfused hearts only increased from the 40^th^ minute after the ischemic insult.

Secondly, our data show that OA exerts cardioprotective effects by attenuating HBP flux in hyperglycemic rat hearts. These data are in agreement with previous work from our laboratory that established that hyperglycemia results in greater oxidative stress, increased BAD *O-*GlcNAcylation and BAD-Bcl-2 dimer formation, thereby mediating HBP-induced myocardial apoptosis in H9c2 cardiomyoblasts [13]. Thirdly, it is also possible that the OA-mediated decrease in oxidative stress may also limit hyperglycemia-induced inactivation of the sarco/endoplasmic reticulum Ca^2+^-ATPase [51] and electrophysiological alterations (arrhythmias, QT prolongation) [47], [52], [53]. Thus our study shows that OA exerts anti-oxidant effects that attenuate myocardial apoptosis and thereby improve contractile functional recovery following ischemia-reperfusion of hyperglycemic rat hearts.

Our *ex vivo* pre-ischemic data suggest that the cardioprotective effects of OA are not dependent on any inherent inotropic effects, but rather involve anti-oxidant and anti-apoptotic mechanisms. The potent hypotensive properties of OA confirm previous observations in Dahl salt sensitive [23] and STZ-diabetic rats [21]. These data therefore offer significant clinical promise considering that hypertension is a robust risk factor for diabetes-induced CVD and non-diabetic heart diseases. The mechanisms underlying this interesting phenomenon were, however, not elucidated in this study. It is possible that OA treatment may also act to modulate the nervous system, but further studies are required to prove this.

We next investigated the role of the UPS in hyperglycemic hearts exposed to ischemia-reperfusion since it is the major non-lysosomal pathway for degradation of ubiquinated proteins [54]. We reasoned that since UPS dysregulation occurs during ischemia-reperfusion, it may be an important contributing factor to reduced contractile function found under these conditions [54]. However, conflicting data have been published with both inhibition and activation of the UPS linked to improved heart function in response to ischemia–reperfusion [reviewed in 55,56]. It is likely that the discordant data may be due to differences in experimental models and protocols employed. We detected diminished proteasomal activities in 11 mM perfused rat hearts following ischemia (versus pre-ischemic hearts) (data not shown). By contrast, high glucose perfused hearts exhibited a robust upregulation of trypsin-like, chymotrypsin-like and caspase-like proteasomal activities following ischemia (Figure 8). We propose that ischemia-reperfusion together with hyperglycemia trigger greater oxidative stress that may result in increased misfolded proteins that are targeted for removal by UPS degradation. However, excessively high UPS activation may result in the activation of damaging signaling pathways, e.g. nuclear factor-kappa B (NFκB) [10]. Here greater UPS activity will degrade IκB (NFκB inhibitor) and release unbound NFκB to the nucleus to induce expression of various genes that may exacerbate inflammation and reperfusion injury [57], [58]. In support of this concept, Pye et al. [16] ascertained that proteasomal inhibition blunted NFκB activation during reperfusion, thereby resulting in decreased reperfusion injury. Moreover, hyperglycemic rats treated with bortezomib (protease inhibitor) exhibited diminished UPS, inflammation and myocardial damage in response to ischemia-reperfusion [10]. In agreement, we found that OA treatment attenuated proteasomal activities in high glucose hearts following ischemia-reperfusion. Together our data show that hearts exposed to high glucose levels and subjected to ischemia-reperfusion display UPS overactivity that may impair the heart’s functional recovery. Moreover, we established that OA treatment inhibits the UPS and that this is linked with improved cardiac contractile function following ischemia-reperfusion under simulated hyperglycemic conditions.

How do our data differ from previous studies that implicate glucose-insulin-potassium (GIK) in cardioprotection? We propose that the protective effects of GIK largely depend on the actions of insulin i.e. via a) its direct cardioprotective (anti-apoptotic) effects [58]–[61] and b) its glucose lowering abilities. It is well established that insulin administration should increase plasma glucose clearance thus limiting damaging effects of acute and chronic hyperglycemia (‘glucotoxicity’). Moreover, insulin promotes glucose uptake and the generation of glycolytic ATP that is linked with cardioprotection [61]. GIK treatment has resulted in mixed success, e.g. treatment of patients with acute myocardial infarction (CREATE-ECLA trial) did not result in any significant cardioprotective effects [62]. Here it is proposed that the potential protective effects of GIK may have been abolished by higher blood glucose levels in GIK-treated patients [58]. Finally, a recent study investigating glucose-insulin treatment in patients undergoing coronary artery bypass grafting (but while maintaining normoglycemia) found that it was cardioprotective and improved myocardial function [63]. Together these data strongly indicate that insulin acts as the major protective component of GIK, and that higher blood glucose levels are in fact damaging to the cardiovascular system.

In summary, this study demonstrates that OA acts as a novel cardioprotective agent in hearts exposed to high glucose levels by decreasing oxidative stress, reducing apoptosis and the UPS, leading to cardioprotection following ischemia-reperfusion. These data are promising since it may eventually result in novel therapeutic interventions to treat stress-induced acute hyperglycemia (in non-diabetic patients) and also diabetic patients with associated CVD complications, e.g. acute myocardial infarction. We are of the opinion that this is particularly relevant within the developing world context, where it may provide a cost-effective therapeutic intervention for the treatment of acute myocardial ischemia in these individuals.
